# 

**Published:** 1888-01

**Authors:** 


The Chicago Medical Journal and Examiner.

Volume 56. Number 1.

### CONTENTS OF THIS NUMBER.

**Original Communications—** PAGE.

A New Method of Treating the Vegetable Parasitic Diseases of the Skin. H. J. Reynolds ...... 1
Two Cases of Abdominal Surgery. R. G. Bogue ...... 4

**Editorial—**

Medical Investigation and Its Practical Results ...... 6
Medical Appointments in Public Hospitals ...... 7
Medical Legislation ...... 7

**Society Reports—**

CHICAGO GYNÆCOLOGICAL SOCIETY—

Vaginal Hysterectomy. J. H. Etheridge ...... 10
Carcinoma of the Cervix. C. Fenger ...... 13

OPHTHALMOLOGICAL SOCIETY OF THE UNITED KINGDOM—

An Unusual Complication after Subconjunctival Tenotomy of the External Rectus. A. Emrys-Jones 19
Persistent Hæmorrhage in the Anterior Chamber after Iridectomy for Chronic Glaucomy. A. Emrys-Jones ...... 19
Congenital and Hereditary Defect of Ocular Movements. Mr. Lawford ...... 19
Exostosis in Orbit. Mr. Silcock ...... 20
Dotted Fundus in Night-Blindness. Mr. Nettleship ...... 20

MEDICO-CHIRURGICAL SOCIETY OF MONTREAL—

Parasitic Onyc ia. Dr. Bell ...... 21
Broncho-Pneumonia. Dr. Johnson ...... 21
Amputation of Thigh for Periosteal Sarcoma. Dr. Bell ...... 21
Osteotomy for Bow-Legs. Dr. Bell ...... 21
Acetanilide. Dr. McConnell ...... 22

MEDICAL SECTION OF THE ROYAL SOCIETY OF NEW SOUTH WALES—

A New Spirometer and Dynamometer ...... 23
Hair-Pin from the Urethra of a Girl ...... 23
Condyloma of Vulva ...... 23
Removal of Uterus and Appendages for Uterine Fibroid ...... 23
Calculus formed around a Hair-Pin in the Bladder of a Child ...... 23
Gangrene of Finger from Pure Carbolic Acid ...... 23
Amputation at Hip Joint, necessitated by "Caisson Fever." Dr. Twynam ...... 24
Marked Case of Curvature of Left Tibia. Dr. MacCulloch ...... 24
Case of Epithelial Growth, with Colloid Degeneration. Dr. Sydney Jones ...... 24
A Case of Gastrotomy for Œsophageal Stricture. Dr. Chisholm ...... 24
A Case of Vicious Union of the Tibia. Dr. Waugh ...... 24
A Case of Excision of the Rectum. Dr. Goode ...... 24
A Case of Laparotomy for Intestinal Obstruction—Recovery. Dr. Mander Jones ...... 24

**Foreign Correspondence—**

London Letter. Dr. W. B. Meany ...... 25

**Abstracts and Extracts—**

Report of the Surgeon-General of the United States Army, 1887 ...... 28
Report of the Supervising Surgeon-General of the United States Marine-Hospital Service, 1887 ...... 36
Ptomaines. C. O. Curtman ...... 41
The Pupil in Health and Disease. J. Chase ...... 47
The Pupil as a Guide in the Administration of Chloroform. H. J. Nelson ...... 49
"Word-Blindness" ...... 50
Extirpation of the Larynx ...... 51
The Medical Corps of the Navy ...... 53
The Action of Drugs on the Kidney ...... 54
Renal Stone and its Diagnosis ...... 55
The Pathology of Old Age ...... 56
The Use of Water at Meals ...... 57
Radical Cure of Hernia ...... 58
Color-Blindness a Brain Affection ...... 59
The Danger of Keeping Dogs ...... 59
The New York Quarantine ...... 59

**Book Reviews** ...... 60

**Books Received** ...... 63

**Items** ...... 64

